# Stem Cell Therapy with Overexpressed VEGF and PDGF Genes Improves Cardiac Function in a Rat Infarct Model

**DOI:** 10.1371/journal.pone.0007325

**Published:** 2009-10-07

**Authors:** Hiranmoy Das, Jon C. George, Matthew Joseph, Manjusri Das, Nasreen Abdulhameed, Anna Blitz, Mahmood Khan, Ramasamy Sakthivel, Hai-Quan Mao, Brian D. Hoit, Periannan Kuppusamy, Vincent J. Pompili

**Affiliations:** 1 The Dorothy M. Davis Heart and Lung Research Institute, The Ohio State University, Columbus, Ohio, United States of America; 2 Cardiovascular Research Institute, Case Western Reserve University, Cleveland, Ohio, United States of America; 3 Arteriocyte, Inc., Cleveland, Ohio, United States of America; 4 Department of Materials Science and Engineering & Whitaker Biomedical Engineering Institute, Johns Hopkins University, Baltimore, Maryland, United States of America; City of Hope National Medical Center, United States of America

## Abstract

**Background:**

Therapeutic potential was evaluated in a rat model of myocardial infarction using nanofiber-expanded human cord blood derived hematopoietic stem cells (CD133+/CD34+) genetically modified with VEGF plus PDGF genes (VIP).

**Methods and Findings:**

Myocardial function was monitored every two weeks up to six weeks after therapy. Echocardiography revealed time dependent improvement of left ventricular function evaluated by M-mode, fractional shortening, anterior wall tissue velocity, wall motion score index, strain and strain rate in animals treated with VEGF plus PDGF overexpressed stem cells (VIP) compared to nanofiber expanded cells (Exp), freshly isolated cells (FCB) or media control (Media). Improvement observed was as follows: VIP>Exp> FCB>media. Similar trend was noticed in the exercise capacity of rats on a treadmill. These findings correlated with significantly increased neovascularization in ischemic tissue and markedly reduced infarct area in animals in the VIP group. Stem cells in addition to their usual homing sites such as lung, spleen, bone marrow and liver, also migrated to sites of myocardial ischemia. The improvement of cardiac function correlated with expression of heart tissue connexin 43, a gap junctional protein, and heart tissue angiogenesis related protein molecules like VEGF, pNOS3, NOS2 and GSK3. There was no evidence of upregulation in the molecules of oncogenic potential in genetically modified or other stem cell therapy groups.

**Conclusion:**

Regenerative therapy using nanofiber-expanded hematopoietic stem cells with overexpression of VEGF and PDGF has a favorable impact on the improvement of rat myocardial function accompanied by upregulation of tissue connexin 43 and pro-angiogenic molecules after infarction.

## Introduction

Recent reports indicate that infusion of hematopoietic stem/progenitor cells is an important therapeutic approach for the treatment of hematological malignant and ischemic diseases [1], [2], [3], [4], [5], [6]. However, insufficient number of donor stem cells, cell viability and inefficient expansion techniques limit its clinical application [1], [2], [3], [4]. Therefore, an efficient and practical *ex vivo* stem cell expansion methodology is critical, which would maintain the expanded stem cell's potential for engraftment, differentiation and long-term sustainability [7]. Stem cells in bone marrow generally maintain the ability to balance self-renewal and commitment to differentiation, whereas in culture, they tend to lose their self-renewal ability after proliferation [7].

Accordingly, to maintain self-renewal ability of stem cells, a stromal-free suspension culture has been rapidly adopted as an alternative to stromal layer culture for stem cell expansion due to their chemically defined nature. The use of various combinations of growth factors and cytokines in suspension culture to substitute for the regulatory signals provided by stromal cells has been utilized [8], [9]. Previous studies from our group and others using stromal-free culture, have shown that the human umbilical cord blood derived CD133+/CD34+ cells can be expanded efficiently on aminated nanofibers for 10 days using serum-free medium [10]. However, their functionality and potential therapeutic efficacy have not yet been clearly defined for treatment of myocardial infarction.

Ischemic heart disease is a major health concern in both the industrialized and developing world. Despite advances in myocardial reperfusion strategies and novel pharmacological approaches, therapies directed towards the deleterious consequences of acute and chronic myocardial ischemic damage remain limited. Myocyte replication and myocardial regeneration have been documented in the human heart after acute myocardial infarction and in chronic ischemic cardiomyopathy. In addition, experimental animal studies and clinical trials suggest that the transfer of stem and progenitor cells into the myocardium has a favorable impact on tissue perfusion and contractile performance [11], which have been corroborated with neovascularization and myocyte formation [12], [13], [14].

Collateral circulation in ischemic heart disease plays a critical role in myocardial ischemia. Recently, novel approaches that may augment collateral circulation in ischemic heart disease have been tested in preclinical and clinical studies. Gene transfer of angiogenic growth factors has been reported to attenuate tissue ischemia through stimulating angiogenesis at sites of neovascularization [15], [16], [17]. Studies with plasmid vectors of vascular endothelial growth factor VEGF-A_164_, or platelet derived growth factor PDGF-BB, or a combination of the two [18] confirmed unique roles of each gene. Capillary density was primarily increased with VEGF-A_164_ while arteriolar growth was preferentially stimulated by PDGF-BB. However, combination of both genes resulted in increase of both capillaries and arterioles in animal models. Furthermore, combined stimulation with VEGF-A, fibroblast growth factor (FGF-2), or PDGF-BB has also emerged as a potent strategy for therapeutic angiogenesis [19]. Circulating CD34+ progenitor cells isolated from the peripheral blood of adult humans [20], [21], [22], represent an alternative approach. There is increasing evidence to suggest that part of the favorable impact of angiogenic growth factor therapy involves the mobilization of bone marrow-derived progenitor stem cells from host cells [23], [24], [25].

In the present study, using *ex vivo* nanofiber scaffold and serum-free expansion technology, we successfully expanded human umbilical cord blood-derived CD133+/CD34+ progenitor cells without promoting differentiation. We further transiently overexpressed angiogenic genes VEGF and PDGF in *ex vivo* expanded stem cells using a bicistronic vector, in which both VEGF-A_164_ and PDGF-BB genes are within the internal ribosomal entry site under CMV promoter, to augment stem cell effect for treatment of myocardial ischemia induced in rat model. The underlying mechanisms of stem cell-therapeutic effect were also investigated.

## Results

### Isolation, Expansion and Genetic Modification of Cord Blood Derived Progenitor Cells

CD133+ cells isolated from freshly collected umbilical cord blood samples using Auto MACS system were of greater than 95% purity. Cells were expanded (225-fold within 10 days) on nanofiber matrices preserving stem cell phenotype [26].

### Transfection of Expanded Progenitor Cells with Coupled VEGF and PDGF

Nanofiber-expanded progenitor cells were transfected, using the Amaxa electroporation protocol described earlier [26]. Transfection efficiency with pmaxGFP vector was more than 90% and cell viability was ∼70%. However, when used VEGF and PDGF containing (VIP) vector transfection efficiency was ∼60%, and cell viability was also ∼60%. Reduced transfection efficiency and cell viability were likely due to the larger plasmid size.

### Angiogenic Factors-Overexpressed Stem Cell Therapy Reduces Myocardial Infarct Area

**Figure 1:**
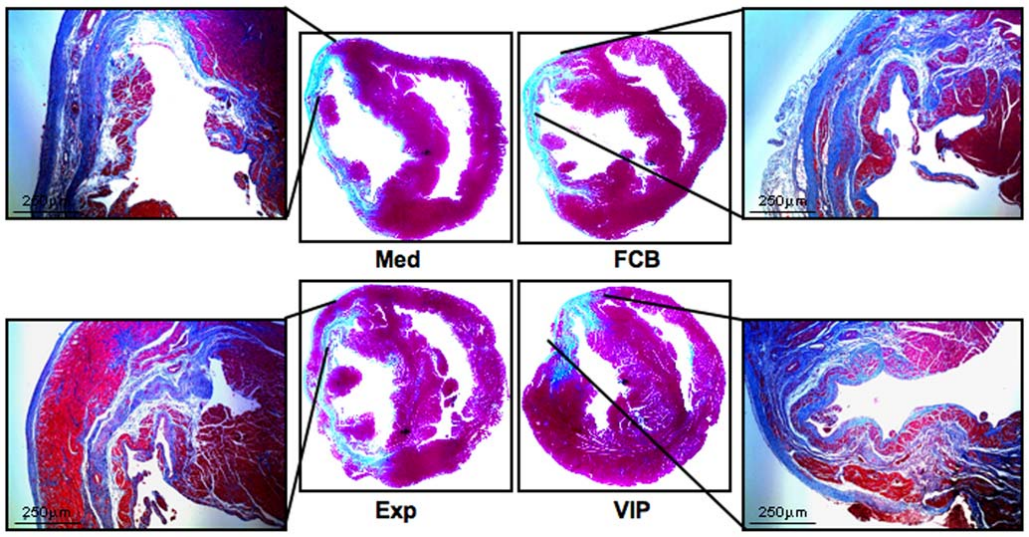
Evaluation of total ischemic area using Masson's trichrome staining. Paraffin-blocked tissues from infarct zones were sectioned and stained with Masson's trichrome after six weeks of therapy demonstrating decreased infarct size in VIP < Exp < FCB < Med groups. Five sections were examined from each heart. Four hearts from each group were evaluated. Every section was examined with low power field to visualize gross heart morphology, and to visualize fibrous tissues after ischemia, and area of interest of each heart section is magnified. Representative gross heart pictures are shown here with areas of interest in higher magnification.

Paraffin blocked sections of harvested rat heart tissue after 6 weeks of stem cell therapy or control groups were stained with Masson's trichome staining and viewed under low powered microscope (Figure 1). Total fibrosis was measured by using UTHSCA Image Tool software (version 3.0). Mean values were reported as percent fibrosis of total heart. Med  = 17.87±1.84, FCB  = 16.10±0.89, Exp  = 15.33±1.43 and VIP  = 11.04±1.82. Animals in the VIP group had reduced fibrous area compared to other groups. A modest reduction in scar was also observed in the Exp group.

### Improved Heart Function after Therapy with Stem Cells Overexpressing Angiogenic Factors

**Figure 2:**
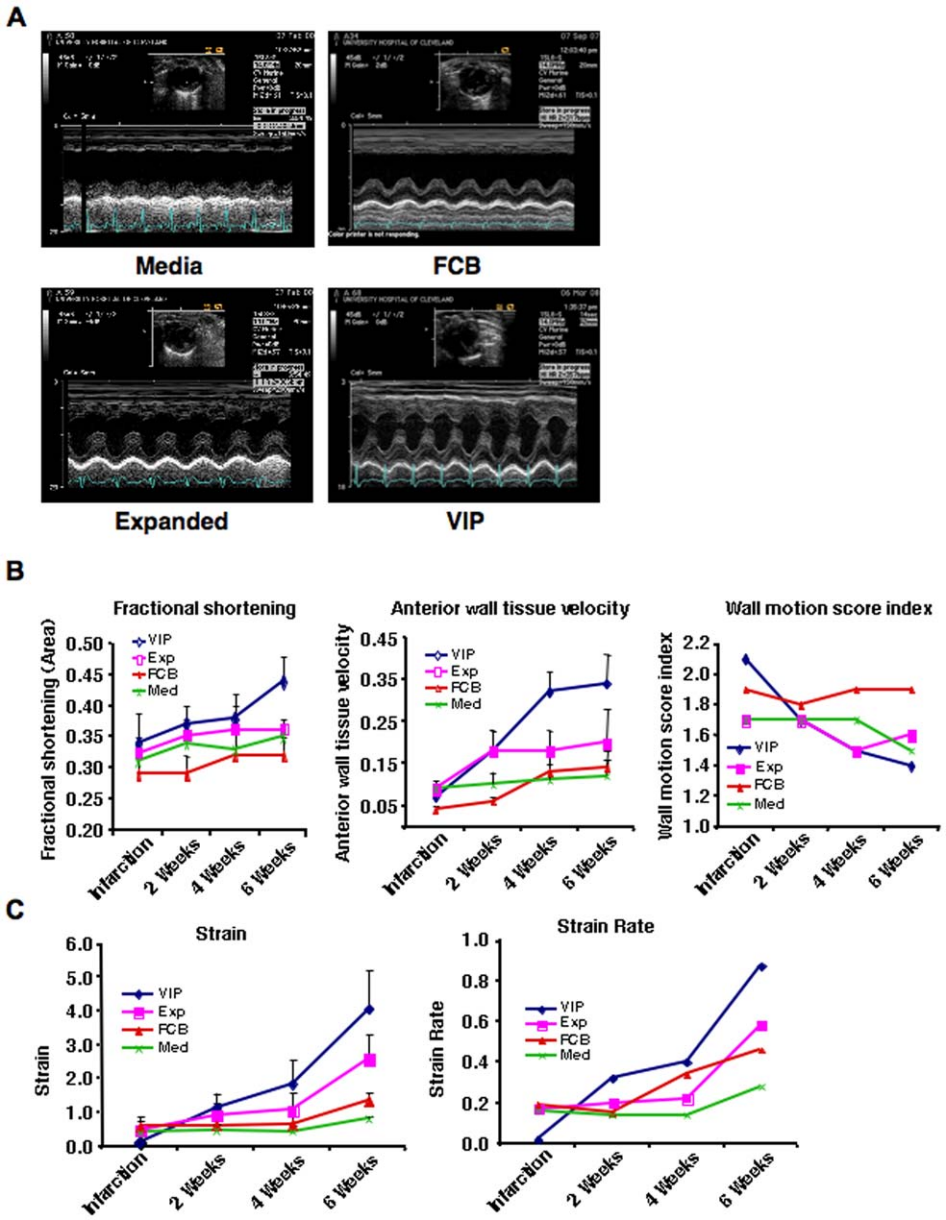
Evaluation of cardiac function by echocardiography. (A) Representative M-mode echocardiographic images after six weeks of therapy demonstrating improved anterior wall motion in the VIP > Exp > FCB > Med groups. Inset shows the short axis two-dimensional image of the LV. (B) Echocardiographic parameters of cardiac function using fractional shortening (FSa), anterior wall tissue velocity (TVa) and wall motion score index (WMSI) plotted as a function of time within each group demonstrating significant improvement in function in the VIP group compared to controls. (C) Strain and strain rates presented as a function of time within each group with significant improvement in the VIP group.

After six weeks of therapy, a remarkable improvement in anterior wall motion by M-mode echocardiography was observed in the VIP group. There was also a remarkable improvement in the Exp group compared to FCB or Med groups (Figure 2A). Other parameters such as fractional shortening (FSa; VIP = 0.44±0.04, Exp = 0.32±0.02, FCB = 0.28±0.03, Med = 0.35±0.05), anterior wall tissue velocity (TVa; VIP = 0.34±0.07, Exp = 0.18±0.08, FCB = 0.14±0.04, Med = 0.12±0.04), and wall motion score index (WMSI; VIP = 1.4±0.1, Exp = 1.6±0.1, FCB = 1.9±0.1, Med = 1.5±0.1) were also markedly improved in the VIP group (Figure 2B) compared to the other groups. Strain (VIP = 4.07±1.15, Exp: 2.57±1.33, FCB: 1.34±0.25, Med: 0.81±0.08), and strain rates (VIP  = 0.87, Exp  = 0.58, FCB  = 0.46, Med  = 0.28) were also increased in a time dependent fashion in the VIP group compared to other groups (Figure 2C).

### Improved Heart Function Correlates with Increased Neovascularization

**Figure 3:**
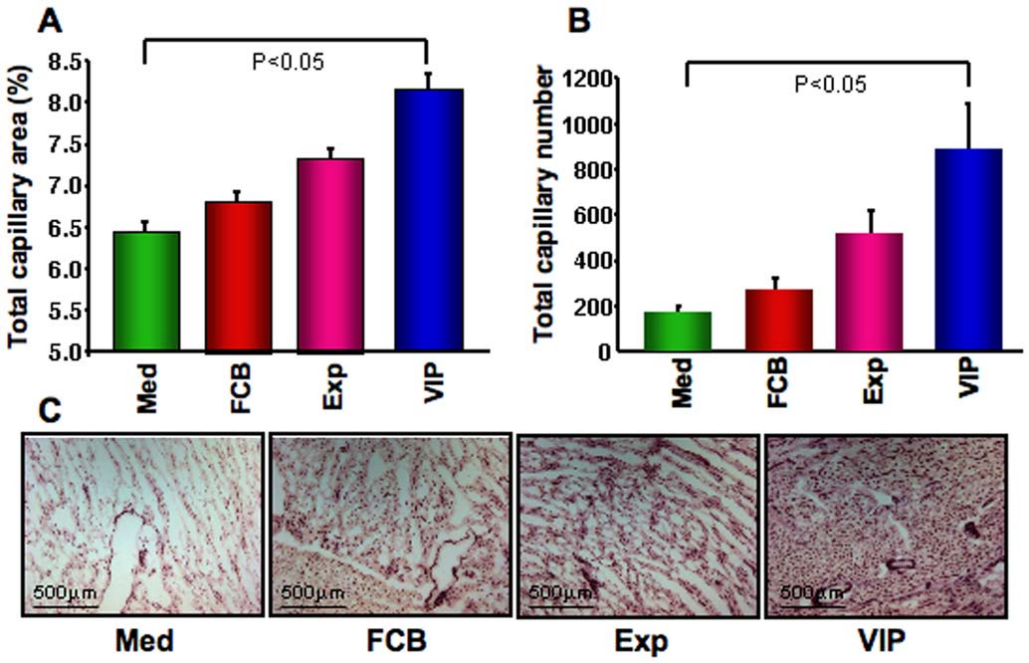
Evaluation of neovascularization. Cyro-preserved sections were stained with alkaline phosphatase, and were evaluated capillary density area (%) per high power field (A), and total capillary number per high power field (B) in histochemistry images. Representative images of each group (C) were shown in the lower panel. Capillary count and total capillary areas (A & B) were measured by Image Quant software demonstrating highest capillary area density in the VIP group.

Capillary density was measured after alkaline phosphatase staining, as described earlier, in the cryo-preserved heart tissue as an anatomical marker of induced angiogenesis. Total percent capillary area per high power field was as follows: VIP = 7.44±0.98, Exp = 7.33±0.91, FCB = 6.82±1.13, and Med = 6.73±0.6. Total capillary was also counted and mean capillary number per high power field is as follows: VIP = 886.0±200.9, Exp = 523.9±100.3, FCB = 272.0±49.6, and Med = 177±25.1. Neovascularization was much more evident in the VIP group compared to other groups, verified by immunostaining and total capillary density (Figure 3).

### Improved Cardiac Function Results in Increased Exercise Capacity on Treadmill

**Figure 4:**
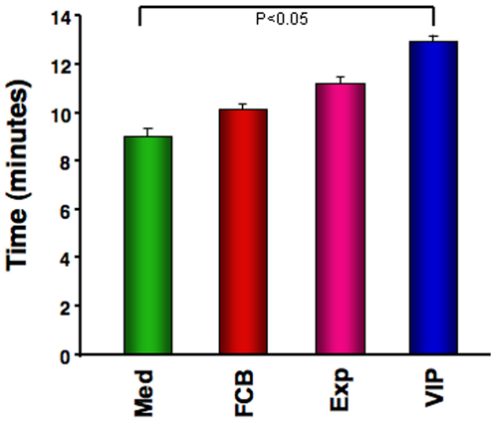
Measurement of exercise capacity. Exercise capacity was evaluated by time on a graded treadmill after six weeks of therapy demonstrated an incremental time in the order of VIP > Exp > FCB > Med groups.

A modest but significant improvement of exercise capacity was observed in animals in the VIP group compared to those in the Med group (Figure 4). A smaller degree of improvement was also observed in the Exp and FCB groups.

### Stem Cells Home to Ischemic Region of the Heart

**Figure 5:**
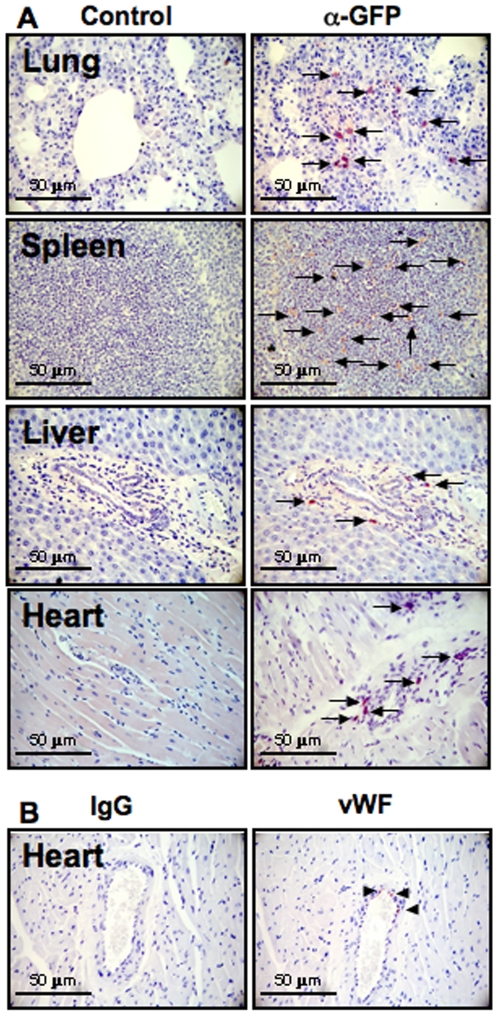
Detection of stem cell homing. (A) Immunohistochemical detection was performed 36 hours after half a million of GFP overexpressed nanofiber expanded stem cells injected into the left ventricle of the rats, with fixed and paraffin embedded tissue sections from various organs using anti-GFP Ab (right panels) against respective controls (left panels) confirming stem cell homing to lung, spleen, liver and heart. (B) Heart sections were stained for human specific von Willebrand factor (vWF) and immunoglobulin G (IgG) as a control.

As demonstrated here, stem cells migrate to lung, spleen and liver (Figure 5A). However, stem cells were also observed to home to ischemic zones of the heart (Figure 5A). Cell numbers were counted per high power field per organ and were as follows: Lung = 21±8 cells, Spleen = 75±12 cells, Liver = 5±2 cells and Heart = 13±4 cells. Migrated stem cells were also found to differentiate into endothelial cells within the arterioles and capillaries in the myocardium (stained with human specific vWF, Figure 5B).

### Upregulation of Gap Junctional Protein Connexin 43

**Figure 6:**
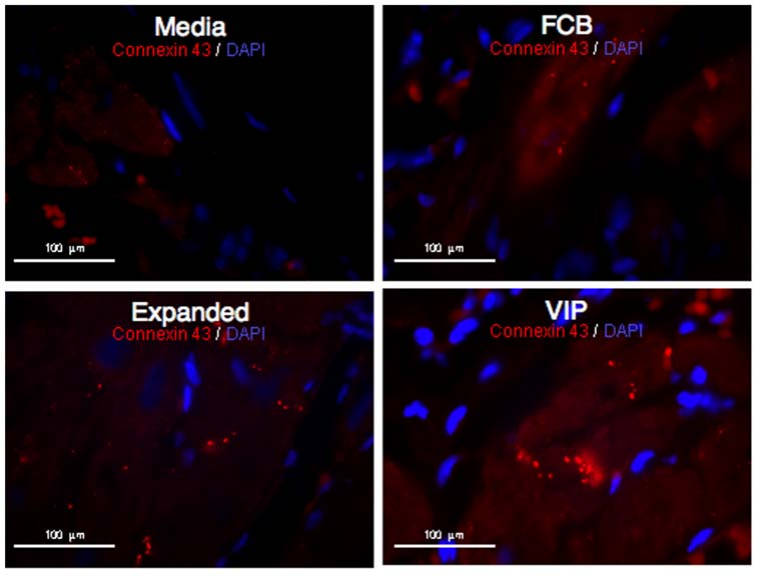
Expression of gap junctional protein, connexin 43. Heart tissue sections were stained for gap junctional protein, connexin 43 from all four groups using immunohistochemistry. Red color indicates connexin 43 stain and blue color indicates DAPI, a nuclear stain. Merged connexin 43 and DAPI of each group is displayed in micrographs.

Gap junctional protein connexin 43, which is a major factor responsible for cardiomyocyte contractile function, was found to have higher expression in the VIP group compared to others (Figure 6). However, modestly increased expression of connexin 43 was also found in animals in the Exp group compared to those in the FCB and Med groups (Figure 6).

### Evaluation of Signals Responsible for Angiogenesis

**Figure 7:**
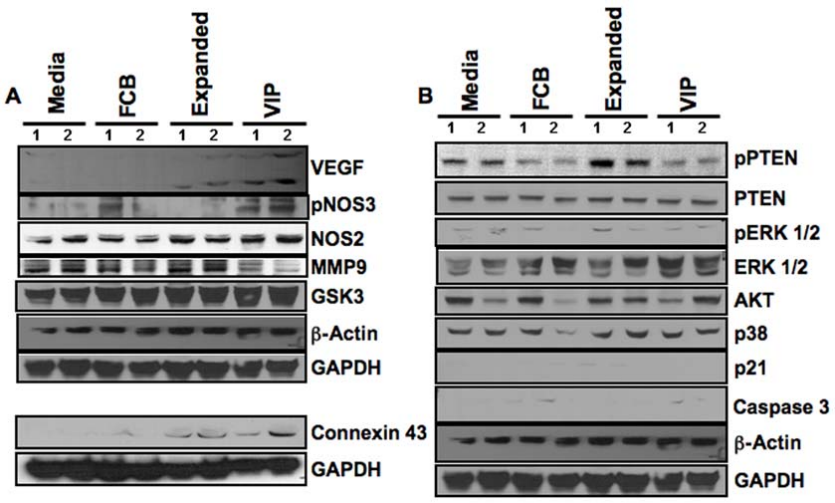
Expression of signaling molecules. (A) Western blot of angiogenesis related and connexin 43 proteins from each animal group. (B) Western blot of various molecules of oncogenic potential and relevant signaling pathways from each animal group.

Total protein isolation and Western blots were performed on snap-frozen heart tissue to assess levels of each individual protein that may play a role in angiogenesis (Figure 7A). Animals in the VIP group had elevated levels of VEGF (9.21±2.19 fold) and phosphorylation of pNOS3 (2.64±0.48 fold) and connexin 43 (4.19±0.57 fold) compared to Med control group. A modest increase was also found in NOS2 (2.21±0.44 fold) and GSK3 (1.44±0.13 fold) expression in the VIP group. A significant reduction of matrix metalloproteinase 9 (MMP 9; 0.53±0.11 fold) was observed in animals treated with VIP compared to others. A modest increase in VEGF (2.06±0.24 fold) and connexin 43 (3.05±0.55 fold) levels were also found in the Exp group compared to Med control group. GAPDH and β-actin were used as internal loading controls.

### Evaluation of Molecules of Oncogenic Potential and Relevant Signaling Pathways

The oncogenic potential of angiogenic factor overexpression was evaluated with no significant inductions in the levels of PTEN, pPTEN, ERK1/2, pERK ½, AKT, p38, p21 and caspase 3 in heart tissues of animals in the VIP group compared to others (Figure 7B).

## Discussion

The availability of adequate quantity of functional hematopoietic stem cells for functional dose-response is one of the major limitations of regenerative cell based therapy. However, our nanofiber-mediated expansion technology provides a unique opportunity to overcome this deficiency. We have previously shown that freshly isolated CD133+ stem cells can be expanded 225-fold in 10 days after culturing them in a serum-free medium on a nanofiber scaffold [26]. We have also demonstrated that these expanded cells retain their progenitor cell phenotypes with respect to multipotential differentiation and migration [26]. As it is not known whether these nanofiber-expanded stem cells are biologically active to be used clinically for the treatment of myocardial ischemia, we sought to investigate the functional role of these cells in a rat myocardial infarction model.

Cell based therapeutic approaches may prevent deterioration of the myocardial function post infarction and may even reverse established heart failure. However, the original concepts regarding cell-based therapies have proven to be overly simplistic since generation and engrafment of new muscle alone is insufficient for the approach to be successful. It appears that nonmyogenic mechanisms are important for majority of the beneficial trophic effects observed during cell-based therapies [27], [28]. Therefore, elucidation of these specific mechanisms is essential to improve cell-based therapies.

In the present study, we first expanded stem cells *ex vivo* on nanofiber-coated plates and then overexpressed VEGF and PDGF in these cells to enhance their vasculogenic potential [29], [30], [31]. Previous studies of autologous BM-derived CD133+ cells injected either *via* intracoronary infusion [32] or intramyocardial injection [33] augmenting vasculogenesis in patients with myocardial ischemia [34] prompted us to investigate the potential of genetically modified stem cells in ischemic heart disease. Furthermore, the limited availability of functional progenitor cell population in bone marrow and peripheral circulation [35] and compromised potential of these cells in disease states and aged individuals [36], [37] has hindered the study of underlying mechanisms of successful cell-based therapy in these subjects. Our current findings thus provide the feasibility and effectiveness of overexpressing angiogenic growth factors on nanofiber expanded hematopoietic stem cells for the treatment of myocardial infarction. The overexpression of angiogenic factors not only promotes neovascularization in the ischemic heart tissues, but thereby significantly improves several parameters of cardiac function including fractional shortening, tissue velocity, wall motion score index, strain and strain rate. Accordingly, with improved heart function, animals demonstrate better exercise capacity implying functional improvement. In addition, the reduction in myocardial fibrosis in these animals is also an important indicator for improved heart function since late reperfusion of infarcted vascular beds attenuates left ventricular remodeling including infarct expansion [38], [39].

In addition to myogenesis, vasculogenesis [40] and anti-apoptotic effects of the paracrine factors secreted by hematopoietic stem cells [41], our findings also indicate that the hematopoietic stem cells home to injured sites besides their usual destinations such as lung, liver, spleen and bone marrow. This phenomenon therefore suggests that both trans-differentiation and paracrine mechanisms might be operational in our therapeutic approach. Nanofiber expanded stem cells express significantly more CXCR4 and high concentrations of SDF-1 in ischemic or damaged tissues, which may be responsible for increased homing as CXCR4 is a natural receptor for SDF-1 [42], [43]. As we report, hematopoietic stem cells home to the injured heart besides the usual destinations such as the lung, liver, spleen and bone marrow. In this homing of stem cells both transdifferentiation and paracrine mechanisms might be involved. In cardiac tissues, infused stem cells differentiate into endothelial cells and integrate into the inner lining of microvasculature under influence of microenvironment. We have previously demonstrated that nanofiber expanded stem cells maintain the ability to multi-potentially differentiate into endothelial and smooth muscle cells, hence, contributing to vasculogenesis [26]. A recent report indicated that hematopoietic stem cells integrated into rat myocardial ischemic tissues and organs could be detected even one month after injection [44]. The involvement of paracrine mechanisms to mediate the therapeutic effects of stem cells in various ischemia models have also been demonstrated [45]. In murine ischemic models, bone marrow stromal cells have been shown to enhance collateral flow recovery and remodeling, thereby attenuating muscle atrophy [46]. Moreover in rat myocardial infarction models, collaterals and improved cardiac function have been demonstrated using CD34+ stem cell therapy [40]. However, our approach of combining angiogenic growth factors and overexpressing them in nanofiber expanded stem cells, resulted in significant enhancement of neovascularization and improvement of cardiac function compared to stem cell injection alone. In addition, to the pro-angiogenic effect of VEGF, this marked improvement of neovascularization may also be due to enhanced maturating effect [29], [47] and inhibition of apoptosis by PDGF [48].

To further elucidate the precise mechanisms of the improved effects of angiogenic growth factors overexpressed in nanofiber expanded stem cells on neovascularization and cardiac function, we evaluated the role of signaling molecules in ischemic rat hearts. We observed a significant increase in the production of VEGF, pNOS3 and NOS2 and decrease in the production of MMP9 in rat heart tissues treated with stem cells overexpressing VEGF and PDGF genes. Since the contribution of angiogenic factors from transfected genes are limited, this type of transfection does not sustain greater than two weeks and only transiently produces these gene products. We, in our current study, noted a similar lower degree of tissue expression of VEGF and NOS2 in tissues harvested after six weeks of stem cell therapy in the Exp group, thereby indicating that stem cell therapy itself induces host tissue production of angiogenic factors, which in turn leads to neovascularization of ischemic tissues. In addition, animals in the VIP group had enhanced tissue production of angiogenic factors, which may have been due to initiation of the signaling cascade and initial effects of VEGF and PDGF produced by the stem cells, further contributing to angiogenesis in the host tissue.

Finally, the concern that transient overexpression leads to tumor formation was addressed by testing for molecules of oncogenic potential and their signaling pathways. As we did not detect enhanced production of these molecules or their signaling pathways of oncogenesis, these results are encouraging for potential clinical use of nanofiber-expanded stem cells.

In summary, we have demonstrated that significantly increased expansion of cord blood-derived stem cells is possible using functional nanofibers and serum-free culture media. These expanded stem cells retain their progenitor cell phenotype and provide an opportunity to genetically manipulate them for cell-based therapy. We have also shown that non-viral delivery of pro-angiogenic factors VEGF and PDGF markedly enhanced the angiogenic effects of stem cells by increasing tissue expression of connexin 43 and growth factors related to angiogenesis. This study thus confirms the feasibility of combined stem cell therapy with pro-angiogenic gene therapy. Furthermore, it also identifies the therapeutic potential of nanofiber-expanded stem cells in treating ischemic heart disease.

## Materials and Methods

### CD133+ Cell Isolation

Fresh human cord blood was obtained from University Hospitals Case Medical Center and The Ohio State University Medical Center after IRB approval and written consent from donors. Cord blood was processed following the similar protocols published previously [26], [49]. In brief, heparinized cord blood was diluted with PBS and carefully layered over Ficoll-Paque. After 30 minutes of centrifugation in a swinging bucket rotor at 1400 rpm, the upper layer was aspirated and the mononuclear cell layer was collected. After several washes with PBS, mononuclear cells were labeled with anti-CD133 monoclonal antibody conjugated to magnetic beads (Miltenyi Biotec Inc, Bergisch Gladbach, Germany). CD133+ cells were isolated by using AutoMACS cell sorter system (Miltenyi Biotec) using double columns, following the manufacturer's protocol and reagents. After separation, purity of the cell product was determined by flow cytometry.

### Electrospinning, Surface Grafting and Amination of PES Nanofiber Mesh

Electrospinning of poly-acrylic acid (PAAc), grafting onto poly-ethyl sulfone (PES) nanofibers (MW: 55,000; Goodfellow Cambridge Limited, UK) and amination were carried out according to the procedure previously described [10].

### Ex-Vivo CD133+ Hematopoietic Cell Expansion

Nanofiber mesh was secured to the bottoms of each of the 24 wells of a tissue culture plate. A total of eight hundred CD133+ cells were seeded onto each scaffold in 0.6 ml StemSpan™ serum-free expansion medium (SFEM; Stem Cell Technologies, Vancouver, BC, Canada), which consists of 1% BSA, 0.01 mg/ml recombinant human insulin, 0.2 mg/ml human transferrin, 0.1 mM 2-mercaptoethanol and 2 mM l-glutamine in Iscove's MDM, supplemented with 0.04 mg/ml low-density lipoprotein (Athens Research and Technology Inc., USA), 100 ng/ml recombinant human stem cell factor (SCF; Peprotech Inc., Rocky Hill, NJ, USA), 100 ng/ml Flt-3 ligand (Flt3; Peprotech Inc), 50 ng/ml Thrombopoietin (TPO; Peprotech Inc) and 20 ng/ml IL-3 (Peprotech Inc). Cells were cultured at 37 °C in an atmosphere containing 5% CO_2_ without change of medium for 10 days. Cells were harvested after 10 days of expansion by washing once with non-trypsin cell dissociation solution and twice with 2% FBS Hanks' buffer at 5–10 minutes intervals between each wash. The cell suspensions collected were then concentrated through centrifugation at 500 × g for 10 min. Aliquots of the concentrated cells were then used for cell counting by a hemocytometer, flow cytometry analysis, as well as for further studies.

### Genetic Overexpression of Angiogenic Factors on Expanded Stem Cells

Nanofiber-expanded stem cells were transfected with green fluorescent protein (GFP)-containing vector (pmaxGFP) and VIP vectors (VEGF-IRES-PDGF in pAMFG vector; gift from Dr. Blau, Stanford University, CA) using a human CD34 cell Nucleofector kit (Amaxa Inc., Walkersville, MD) following manufacturer's protocol. In brief, 1–3×10^6^ cells were transfected with 2–4 µg of plasmid DNA in 100 µl of CD34 cell Nucleofector solution using Amaxa nucleoporator programs: U-008 or U-001 (Amaxa Inc., Walkersville, MD). After transfection, cells were cultured with DMEM complete media or as stated for further studies.

### Animal Model of Myocardial Infarct

All animal experiments were performed in accordance with the guidelines published in the “Guide for the Care and Use of Laboratory Animals” (NRC publication), and under the protocols approved by the Institutional Animal Care and Use Committee (IACUC) at Case Western Reserve University and The Ohio State University (University laboratory animal resources, ULAR). Male athymic nude rats (Hsd:RH-rnu rats, Harlan Sprague Dawley, Indianapolis, IN) aged 6 to 8 weeks were anesthetized with intraperitoneal sodium pentobarbital (50 mg/kg). Myocardial infarct was induced by surgical ligation of the left anterior descending (LAD) coronary artery. Immediately before euthanization, rats were injected with an overdose of pentobarbital.

### Transplantation of Expanded and Genetically Modified Stem Cells

Five days after myocardial infarct, rats were divided into groups (N = 6–9/group) that received intraventricular injections of 5×10^5^ cells that were freshly isolated (FCB), nanofiber-expanded only (Exp), or nanofiber-expanded and angiogenic factors overexpressed (VIP) resuspended with 300 µL of media. The control group received the same volume of media without cells (N = 6–9). Rats were sacrificed 6 wks after intraventricular injection.

### Histological Assessment of Transplanted Animals

At necropsy, hearts were sliced in a bread-loaf fashion into 8 transverse sections from apex to base and fixed with 4% paraformaldehyde. One half of each group of rat tissues was fixed and embedded in OCT compound (Miles Scientific) and the other half snap-frozen in liquid nitrogen for fluorescence microscopy and alkaline phosphatase staining for capillary count. Paraffin-embedded tissues were used to measure the average ratio of fibrosis area to LV area. Capillary density was evaluated morphometrically by histological examination of sections recovered from segments of LV myocardium, subserved by the occluded LAD. Morphometric studies were performed by a single examiner, who was blinded to treatments.

### Tracking of Stem Cell Homing

To assess the homing of stem cells, GFP overexpressed nanofiber expanded stem cells (5×10^5^ cells/rat) were injected into the rat left ventricle five days after induction of myocardial ischemia. Thirty-six hours after cell injection, rats were sacrificed and tissues harvested. Tissues were fixed in 10% formalin solution and subsequently embedded in paraffin block. Five-micron sections were cut and stained using anti-GFP antibody conjugated with alexa fluor dye. After several washing steps, samples were viewed under high power microscopy (Leica). Untransfected NIH3T3 cells and GFP transfected NIH3T3 cells were used as negative and positive controls, respectively.

### Echocardiographic Analysis of LV Function

Rats were anesthetized with 3% isoflurane (maintained at ∼1% by nosecone) in a gas chamber. After shaving the chest, the extremities were secured to a warming pad (Braintree Scientific, MA) with paper tape and needle electrodes connected to a preamplifier to simultaneously record a single lead electrocardiogram. Pre-warmed ultrasound transmission gel was applied to the chest and 2D-directed M-mode and Doppler echocardiographic studies were performed using a 15 MHz (15L8) linear array transducer (Sequoia, Siemens, Mountain View, CA). Rats were imaged in the shallow left lateral decubitus position and short and orthogonal long axis and apical views were obtained. Echocardiography was performed at baseline, 2, 4, and 6-weeks after stem cell injection by an echo technician, who was blinded to the therapy. Data were analyzed using software resident on the ultrasonograph by an experienced researcher, who was also blinded to treatment.

### Measurement of Area Fractional Shortening

Area fractional shortening (FSa) was determined in both the long axis and short axis views as the difference between the LV diastolic area (LVDa) and LV systolic area (LVSa) divided by the LV diastolic area (LVDa) such that FSa  =  (LVDa−LVSa)/LVDa averaged between the long axis and short axis views.

### Measurement of Wall Motion Score Index

LV wall motion score index (WMSI) was analyzed with a 13-segment model that has previously been validated [50]. The wall segments were visualized from two-dimensional images taken from the parasternal long axis and from the basal and midpapillary short axes. Regional wall motion was graded in each segment according to the scheme adopted by the American Society of Echocardiography, where 1 is normal, 2 is hypokinetic, 3 is akinetic, 4 is dyskinetic, and 5 is aneurysmal [51]. Motion in the anteroseptal and posterior wall segments was scored from the clearer of the parasternal long or short axis, but not both. WMSI was defined as the total of the wall motion scores divided by the number of segments scored.

### Measurement of Anterior Wall Tissue Velocity, Strain and Strain Rate

Anterior wall tissue velocity (TVa), strain (TS), and strain rate (TSR) were determined using Velocity Vector Imaging (VVI, Syngo). VVI is a visual and quantitative method for assessing cardiac mechanics using the 2D information within the image that is based on speckle and endocardial border tracking to determine the tissue motion throughout the cardiac cycle. The end-systolic endocardial to mid-wall from the short axis at the papillary muscle was manually traced from a digital cineloop frame. The velocity vector of every point in the border is given by summation of the border motion plus the relative velocity of the tissue with respect to the border. Regions of interest such as the anterior wall (area of infarct) were selected to obtain tissue velocity (cm/s), strain (%), and strain rate (1/s) from the anterior wall (circumferential).

### Measurement of Functional Exercise Capacity

To evaluate functional capacity, rats were placed on an open treadmill (Exer-3/6, Columbus Instruments). The treadmill incorporates an electrified grid at the rear of the belt to provide motivation. Rats were acclimatized to the treadmill by undergoing a 5-minute run at 5 meters per minute without incline before their exercise test. Subsequently, the rats are subjected to supervised, graded treadmill exercise by staged, 3-minute increases in belt speed alternating with incline. Exhaustion was defined as the rat spending greater than 50% of the time or 10 consecutive seconds on the shock grid. Exercise capacity was evaluated at baseline and 6 weeks after injection.

### Capillary Staining and Counting

To determine the capillary density in the border zone of the ischemic heart, tissues were dissected and snap frozen in liquid nitrogen. Cryo-sections of frozen tissues were stained using an alkaline phosphatase kit (Sigma FAST™ BCIP/NBT (5-Bromo-4-chloro-3-indolyl phosphate/Nitro blue tetrazolium tablets), which were used for the detection of alkaline phosphatase activity and stained as a substrate precipitant. For quantification of positively stained vessels (predominantly endothelium), five sections within the necrosis border zone of each animal were analyzed by an investigator who was blinded with respect to the cell treatment. Capillaries were counted in 12 randomly chosen high-power fields (HPFs) in 5 sections per tissue and 5 animals per group. The results were expressed as total capillary area measured by Image software (Image J, NIH, MD).

### Immunohistochemistry

Gap junctional protein connexin 43, which is a major factor responsible for cardiomyocyte contractile function, was assessed by immunohistochemistry, formaldehyde fixed and paraffin-embedded sections of cardiac tissue harvested after six weeks of stem cell therapy.

### Evaluation of Signaling Molecules in Rat Heart Tissue

After six weeks of stem cell therapy, total protein was isolated from snap-frozen total heart tissues by homogenization in 50 mM Tris-Hcl buffer pH 7.5 containing 150 mM sodium chloride, 0.5% NP-40, 1 mM sodium pyrophosphate, 5 mM sodium vanadate, 1 mM benzamidine, 1 mM sodium fluoride and protease inhibitors. Western blotting was performed to assess the level of each protein stated. GAPDH and β-actin were used as internal loading controls. To analyze Western blot band densities, we used UN-SCAN-IT Gel software (version 6.1, Silk Scientific Corp. Utah, USA). Data provided are as fold-increase or decrease compared to Med control. All data were corrected with respect to GAPDH levels.

### Statistical Analysis

All values are presented as mean±standard error of mean. One-way ANOVA with Scheffe's post hoc test for unequal sample sizes was used to compare numeric data between the four experimental groups. Datasets consisting of two groups only were compared by unpaired Student's t-test. A level of p<0.05 was considered as significant difference.
